# Diverse antimalarials from whole-cell phenotypic screens disrupt malaria parasite ion and volume homeostasis

**DOI:** 10.1038/s41598-018-26819-1

**Published:** 2018-06-11

**Authors:** Adelaide S. M. Dennis, James E. O. Rosling, Adele M. Lehane, Kiaran Kirk

**Affiliations:** Research School of Biology, Australian National University, Canberra, ACT 2601 Australia

## Abstract

Four hundred structurally diverse drug-like compounds comprising the Medicines for Malaria Venture’s ‘Pathogen Box’ were screened for their effect on a range of physiological parameters in asexual blood-stage malaria (*Plasmodium falciparum*) parasites. Eleven of these compounds were found to perturb parasite Na^+^, pH and volume in a manner consistent with inhibition of the putative Na^+^ efflux P-type ATPase PfATP4. All eleven compounds fell within the subset of 125 compounds included in the Pathogen Box on the basis of their having been identified as potent inhibitors of the growth of asexual blood-stage *P*. *falciparum* parasites. All eleven compounds inhibited the Na^+^-dependent ATPase activity of parasite membranes and showed reduced efficacy against parasites carrying mutations in PfATP4. This study increases the number of chemically diverse structures known to show a ‘PfATP4-associated’ phenotype, and adds to emerging evidence that a high proportion (7–9%) of the structurally diverse antimalarial compounds identified in whole cell phenotypic screens share the same mechanism of action, exerting their antimalarial effect via an interaction with PfATP4.

## Introduction

The Medicines for Malaria Venture’s (MMV’s) ‘Pathogen Box’ is a set of 400 structurally diverse ‘drug-like’ compounds with activity against a number of different pathogens (https://www.pathogenbox.org/; accessed 20^th^ April 2017). 125 of the compounds in the Pathogen Box were selected on the basis of their ability to inhibit the proliferation of asexual blood-stage *Plasmodium falciparum* parasites in ‘whole cell’ phenotypic screens. The remaining 275 compounds have activity against other pathogens.

MMV had previously released the ‘Malaria Box’, a collection of 400 structurally diverse compounds, all of which had been identified in whole cell phenotypic screens to be effective inhibitors of the proliferation of asexual blood-stage *P*. *falciparum* parasites. Of the 400 compounds, 28 compounds, representing some 16 discrete chemotypes, were shown to have the characteristics of compounds that exert their growth-inhibitory effect through an interaction with the protein PfATP4^1^.

PfATP4 is a member of a distinct subfamily of type II P-type ATPases that is restricted to apicomplexan parasites^2^. PfATP4 has been proposed to function as a plasma membrane Na^+^ efflux ATPase, exporting Na^+^ (thereby maintaining a low cytosolic Na^+^ concentration in the parasite cytosol) whilst importing H^+^ (thereby imposing a significant ‘acid load’ on the parasite)^3,4^. ‘PfATP4-associated’ antimalarials share a number of features in common. Parasites selected for resistance to these compounds are characterised by having mutations in PfATP4^1,5–8^, and parasites selected on the basis of their resistance to one PfATP4-associated compound typically (though not always^6,8^) show cross-resistance to others^1,5,8^. The PfATP4-associated compounds also display a ‘physiological and biochemical phenotype’. The compounds have been reported to cause: (1) an increase in parasite Na^+^ content^9^ and in the parasite’s cytosolic Na^+^ concentration ([Na^+^]_cyt_), attributed to the cessation of active Na^+^ extrusion by the parasite^3–6,8^); (2) an increase in the pH of the parasite cytosol (pH_cyt_) as well as a decrease in the magnitude of the cytosolic acidification seen in response to the addition of the V-type H^+^ ATPase inhibitor concanamycin A to parasites, both attributed to a lifting of the acid load associated with the import of H^+^^1,3–5^; (3) an increase in both parasite cell volume^5,6,10^ and the total volume of the parasitised erythrocyte^10^. Two PfATP4-associated chemotypes have been reported to induce an apparent increase in the cholesterol content of the parasite plasma membrane^11^. The PfATP4-associated compound NITD246 has also been shown to inhibit a Na^+^-dependent ATPase activity associated with the membranes of isolated parasites^4^.

In this study, the Pathogen Box was screened using a number of physiological assays. Eleven compounds in the Pathogen Box were found to show the physiological characteristics of PfATP4-associated antimalarials. All eleven fell within the 125 compounds that had been selected for the Pathogen Box on the basis of their ability to inhibit the proliferation of *P*. *falciparum* parasites in whole cell phenotypic screens. The results add support to the view that selection of antimalarial compounds based on the results of whole cell phenotypic screens yields compound collections in which a significant proportion of the molecules exert their growth-inhibitory effect via an interaction with PfATP4.

## Results

### Multiple compounds in the Pathogen Box disrupt parasite Na^+^ regulation

**Figure 1:**
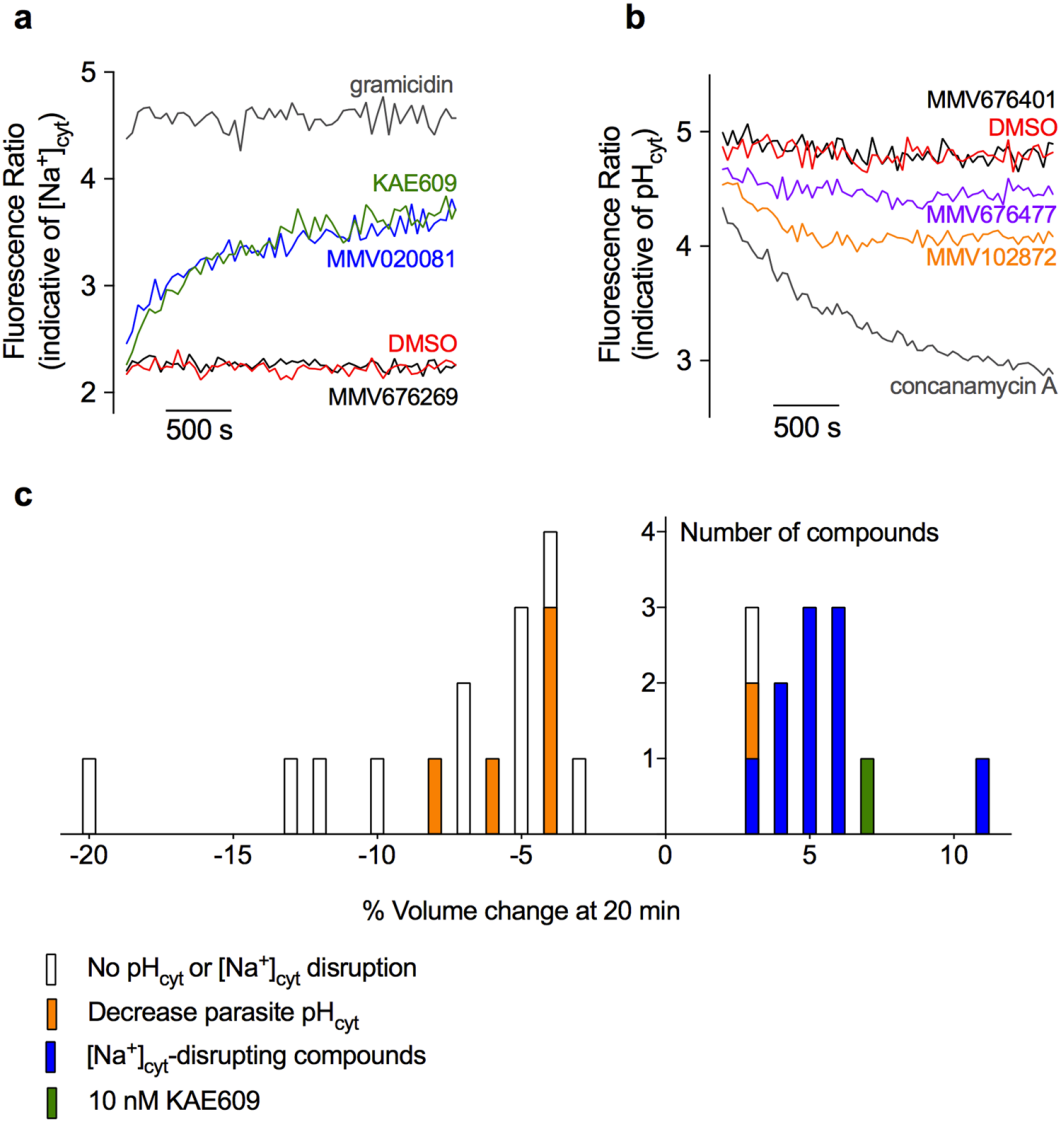
Results of the initial screen of the Pathogen Box for effects on [Na^+^]_cyt_, pH_cyt_ and cell volume in isolated asexual blood-stage 3D7 parasites. (**a**) Representative traces showing the effects of KAE609 (50 nM, green trace), 0.1% v/v DMSO (solvent control, red trace), the Na^+^ ionophore gramicidin (5 µM, grey trace) and two different Pathogen Box compounds (a black trace for the ‘non-hit’ MMV676269 and a blue trace for the ‘hit’ MMV020081, each at 1 µM) on [Na^+^]_cyt_ in isolated SBFI-loaded parasites. For the initial screen each of the four hundred compounds comprising the Pathogen Box was tested for its effect on [Na^+^]_cyt_ a single time (i.e. n = 1). (**b**) Representative traces showing the effects of concanamycin A (100 nM, grey trace), DMSO (0.1% v/v, vehicle control, red trace), and three Pathogen Box compounds (a black trace for the non-hit MMV676401, an orange trace for the hit MMV102872 and a purple trace for the hit MMV676477, each at 1 µM) on pH_cyt_ in isolated BCECF-loaded parasites. For (**a**) and (**b**) parasites were suspended in physiological saline at 1–2 × 10^7^ cells/mL, and compounds were added 5–30 s prior to the start of the measurement. Fluorescence was measured using the Tecan fluorimeter in 96-well plate format. (**c**) The number of Pathogen Box compounds (each tested at 5 µM) that caused a ≥3% change in the volume of isolated trophozoites following a 20 min exposure (n = 2). Pathogen Box compounds that caused an increase in [Na^+^]_cyt_ are shown in blue and KAE609 (10 nM) is shown in green. Pathogen Box compounds that caused a decrease in pH_cyt_ are shown in orange. Parasites were suspended in physiological saline at a density of 4 × 10^5^ parasites/mL.

The initial screen of the Pathogen Box entailed using a fluorescence assay for monitoring parasite [Na^+^]_cyt_, adapted to a 96-well plate format. The PfATP4-associated antimalarial spiroindolone KAE609 (50 nM) and the Na^+^ ionophore gramicidin (5 µM) both served as positive controls. DMSO (0.1% v/v) was added as a (negative) solvent control (Fig. 1a).

Of the 400 compounds comprising the Pathogen Box, eleven (shown in Table 1) caused parasite [Na^+^]_cyt_ to increase when added at a concentration of 1 µM (Fig. 1a). The magnitude of the [Na^+^]_cyt_ increase induced by the eleven compounds varied (Table 1). All of these eleven compounds had been included in the Pathogen Box on the basis of their ability to inhibit the proliferation of asexual blood-stage *P*. *falciparum* parasites in whole cell phenotypic screens. 371 compounds were found to have no effect on parasite [Na^+^]_cyt_ under the conditions tested. For the remaining 18 compounds their addition to the dye-loaded parasite suspension caused an ‘optical effect’; i.e. an abrupt transition of the fluorescence ratio, as arises when the compound itself has intrinsic fluorescent properties or interferes chemically with the SBFI dye and causes a change in the fluorescent output of the dye. For these compounds it was not possible to determine whether they had any effect on [Na^+^]_cyt_.

**Table 1:** Chemical structures of the eleven Pathogen Box compounds found here to cause an increase in [Na^+^]_cyt_ in isolated parasites.

Compound Identifier	Location in Pathogen Box	Structure	Magnitude of [Na^+^]_cyt_ increase (increase in SBFI fluorescence ratio as a % of that induced by KAE609)^**a**^
MMV020623	Plate B, A09		63%
MMV020136	Plate B, C07		68%
MMV020710	Plate B, C08		106%
MMV020520	Plate B, D08		76%
MMV085210	Plate B, F11		21%
MMV006239^b^	Plate B, G07		63%
MMV000858	Plate B, G08		70%
MMV020391	Plate B, H09		43%
MMV020081	Plate D, D03		89%
MMV001059	Plate D, G03		70%
MMV688980	Plate D, H11		27%

^a^The magnitude of [Na^+^]_cyt_ increase for each of the compounds is indicated by the increase in the average fluorescence ratio of SBFI-loaded isolated trophozoites at 24–25 min following the addition of the compound (1 µM), expressed as a % of the increase induced by the addition of the putative PfATP4 inhibitor KAE609 (50 nM).

^b^MMV006239 is structurally similar to the spiroindolone compounds KAE609 (cipargamin) and NITD246.

### Multiple compounds in the Pathogen Box perturb the cytosolic pH (pH_cyt_) of the parasite

The Pathogen Box was also screened for the ability of the compounds to perturb parasite pH_cyt_, again using a 96-well plate format. The V-type H^+^ ATPase inhibitor concanamycin A (100 nM), which causes an acidification of the parasite cytosol by inhibiting the plasma membrane V-type H^+^ ATPase^12^, served as a positive control (Fig. 1b). Ten compounds from the Pathogen Box were found to induce acidification of the parasite cytosol when added at a concentration of 1 µM (Supplementary Table S1). None of these ten compounds were included in the Pathogen Box on the basis of having antiplasmodial activity; all were included in the Pathogen Box on the basis of their effect on other organisms.

The PfATP4-associated antimalarial KAE609 is known to induce an alkalinisation of the parasite cytosol, attributed to a lifting of the acid load associated with the (Na^+^ efflux/H^+^ influx) operation of the transporter^3,4^. When tested using the 96-well plate-based pH assay the KAE609-induced alkalinisation was barely detectable – a consequence of the relatively low sensitivity of this assay. Despite this, nine compounds (MMV020623, MMV020136, MMV020710, MMV020520, MMV006239, MMV000858, MMV020391, MMV020081 and MMV001059) appeared to cause an alkalinisation of the parasite cytosol when added at a concentration of 1 µM, as determined by an overlap of the fluorescence trace with that from parasites to which KAE609 had been added (Supplementary Fig. S1). All nine compounds fell within the eleven compounds identified as being ‘[Na^+^]_cyt_-disrupting’ in the parasite [Na^+^]_cyt_ screen. The other two ‘[Na^+^]_cyt_-disrupting’ compounds, MMV085210 and MMV688980, did not cause a discernible alkalinisation under the conditions tested. Of the eleven ‘Na^+^-disrupting’ compounds, these two compounds – MMV085210 and MMV688980 – caused the smallest increase in [Na^+^]_cyt_ (Table 1) and were the least potent of the eleven compounds at killing parasites, with IC_50_ values (i.e. the concentration of compound required to inhibit parasite proliferation by 50% in a 72 h growth assay) of 1.60 ± 0.07 µM (n = 4) and 1.12 ± 0.07 µM (n = 5), respectively (Table 2). These IC_50_ values are higher than the concentration used in this initial pH screen (1 µM).

**Table 2:** IC_50_ values (i.e. concentrations required to reduce parasite proliferation by 50%) for growth inhibition of the spiroindolone-resistant parasite line NITD609-R^Dd2^-clone#2 and its Dd2 parental line^7^ by the eleven Pathogen Box compounds of interest, the spiroindolone KAE609, and the non-PfATP4-associated antimalarial drugs chloroquine and artemisinin.

Compound	IC_50_ (µM) for inhibition of parasite proliferation				
	Dd2 parent	NITD609-R^Dd2^-clone#2	Fold difference	n	p
MMV000858	0.38 ± 0.02	2.94 ± 0.38	7.7 ± 0.1	4	<0.001
MMV001059	0.72 ± 0.06	3.74 ± 0.31	5.2 0.1	5	<0.001
MMV006239	0.24 ± 0.03	1.87 ± 0.17	7.8 ± 0.1	4	<0.001
MMV020081	0.11 ± 0.01	0.42 ± 0.03	3.8 ± 0.1	5	<0.001
MMV020136	0.52 ± 0.02	3.92 ± 0.28	7.6 ± 0.1	4	<0.001
MMV020391	0.28 ± 0.02	2.76 ± 0.32	9.9 ± 0.1	4	<0.001
MMV020520	0.32 ± 0.04	1.39 ± 0.15	4.3 ± 0.2	4	<0.001
MMV020623	0.37 ± 0.03	2.12 ± 0.31	5.7 ± 0.2	4	0.001
MMV020710	0.098 ± 0.012	0.55 ± 0.03	5.6 ± 0.1	4	<0.001
MMV085210	1.60 ± 0.07	6.8 ± 0.9	4.2 ± 0.1	4	<0.001
MMV688980	1.12 ± 0.07	7.2 ± 0.6	6.4 ± 0.1	5	<0.001
KAE609	0.0011 ± 0.0001	0.012 ± 0.001	11.6 ± 0.1	5	<0.001
Chloroquine	0.21 ± 0.02	0.22 ± 0.02	1.0 ± 0.1	6	0.8
Artemisinin	0.017 ± 0.005	0.019 ± 0.005	1.1 ± 0.4	3	0.8

The IC_50_ values are averaged from those obtained in n experiments and are presented as mean ± SEM. The ‘fold difference’ is the IC_50_ value obtained for NITD609-R^Dd2^-clone#2 divided by the IC_50_ value obtained for the Dd2 parent (shown as the mean ± SEM from the number of experiments indicated). The p values are derived from unpaired student t tests.

### Multiple compounds in the Pathogen Box perturb parasite volume

The Pathogen Box was screened for compounds that perturb the volume of isolated parasites, using the strategy outlined in Supplementary Fig. S2. A compound was deemed a ‘hit’ in the volume assay if it caused the parasite volume to change (i.e. to increase or decrease) by ≥3% following a 20 min exposure. The average volume of isolated parasites was 43 ± 5 fL (mean ± SD; range 34–58 fL; n = 24). A 20 min incubation with the putative PfATP4-antagonist KAE609 (10 nM) caused the parasite volume to increase by 7 ± 2% (mean ± SD; n = 22). Twelve compounds from the Pathogen Box caused the parasite volume to increase by ≥3% when added at a concentration of 5 µM (Fig. 1c; Supplementary Table S1), with the increase in the range 3–11%. Of these 12 compounds, ten had been identified as Na^+^-disrupting compounds in the [Na^+^]_cyt_ screen (Fig. 1c; blue bars). The single remaining [Na^+^]_cyt_-disrupting compound, MMV688980, caused a volume increase of 0.9 ± 0.3% (mean ± range/2, n = 2) at the 5 µM concentration tested here, below the 3% volume change threshold used to identify hits in this screen. Of the two remaining compounds that induced cell swelling, one caused parasite acidification (Fig. 1c; orange bar) and one had no significant effect on either [Na^+^]_cyt_ or pH_cyt_ (Fig. 1c; unfilled bar).

Sixteen compounds from the Pathogen Box (each added at a concentration of 5 µM for 20 min) caused the volume of isolated parasites to *decrease* by ≥3% (Fig. 1c; Supplementary Table S1). The volume decrease ranged from 3–20%. Of these 16 compounds, five were identified as causing a cytosolic acidification in the pH_cyt_ screen (orange bars). The remaining nine compounds had no effect on either parasite pH_cyt_ or [Na^+^]_cyt_ (unfilled bars).

### Eleven Pathogen Box compounds disrupt parasite [Na^+^]_cyt_, pH_cyt_ and parasite volume, inhibit Na^+^-dependent ATPase activity, and show reduced potency against parasites with KAE609-resistance conferring mutations in PfATP4

**Figure 2:**
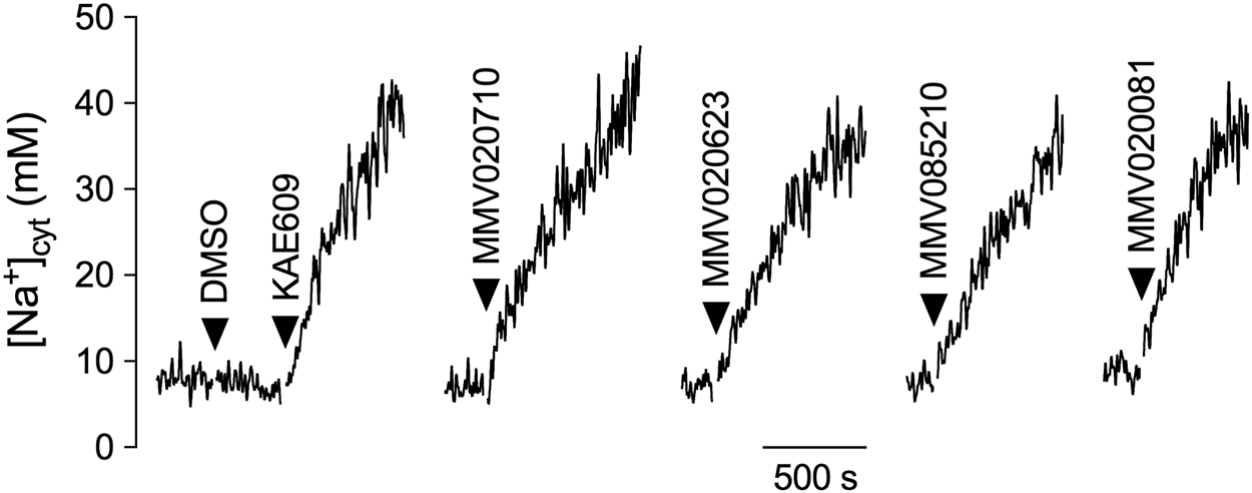
Effect of Pathogen Box compounds on [Na^+^]_cyt_ of isolated asexual blood-stage 3D7 parasites. Representative traces showing the effects of selected compounds on [Na^+^]_cyt_ in isolated SBFI-loaded parasites. DMSO (0.1% v/v), added as a solvent control, had no effect on parasite [Na^+^]_cyt_, whereas the addition of KAE609 (50 nM, added as a DMSO solution) caused an immediate-onset increase in [Na^+^]_cyt_. The addition of the Pathogen Box compounds MMV020710 (1.47 µM), MMV020623 (5.55 µM), MMV085210 (24 µM) or MMV020081 (1.65 µM) caused parasite [Na^+^]_cyt_ to increase. The concentrations tested were, in each case, 15× the IC_50_ for inhibition of parasite proliferation as determined in the laboratory Dd2 strain (Table 2). Parasites were suspended in physiological saline at 1–2 × 10^7^ cells/mL, and fluorescence intensity was measured using a PerkinElmer LS 50B fluorimeter. The results shown are from a single experiment, representative of those obtained in at least three independent experiments.

The eleven compounds identified in the initial [Na^+^]_cyt_ screen as disrupting parasite [Na^+^]_cyt_ regulation (Table 1) were selected for further characterisation. The effect of each on parasite [Na^+^]_cyt_ homeostasis was first confirmed in SBFI-loaded isolated parasites using a PerkinElmer LS 50B fluorimeter. The resting [Na^+^]_cyt_ was 10 ± 3 mM (mean ± SD; n = 6) and the addition of DMSO (0.1% v/v; vehicle control) was without effect (Fig. 2). As was found in the 96-well plate assays, the addition of KAE609 (50 nM in DMSO) caused an immediate-onset, progressive increase in [Na^+^]_cyt_. Each of the eleven compounds of interest caused a similar rise in parasite [Na^+^]_cyt_ when tested at a concentration 15× the IC_50_ for inhibition of proliferation as determined in the laboratory Dd2 strain (Table 2; see Fig. 2 for sample traces) and also caused an increase in [Na^+^]_cyt_ when tested at 1 µM (Supplementary Fig. S3).

**Figure 3:**
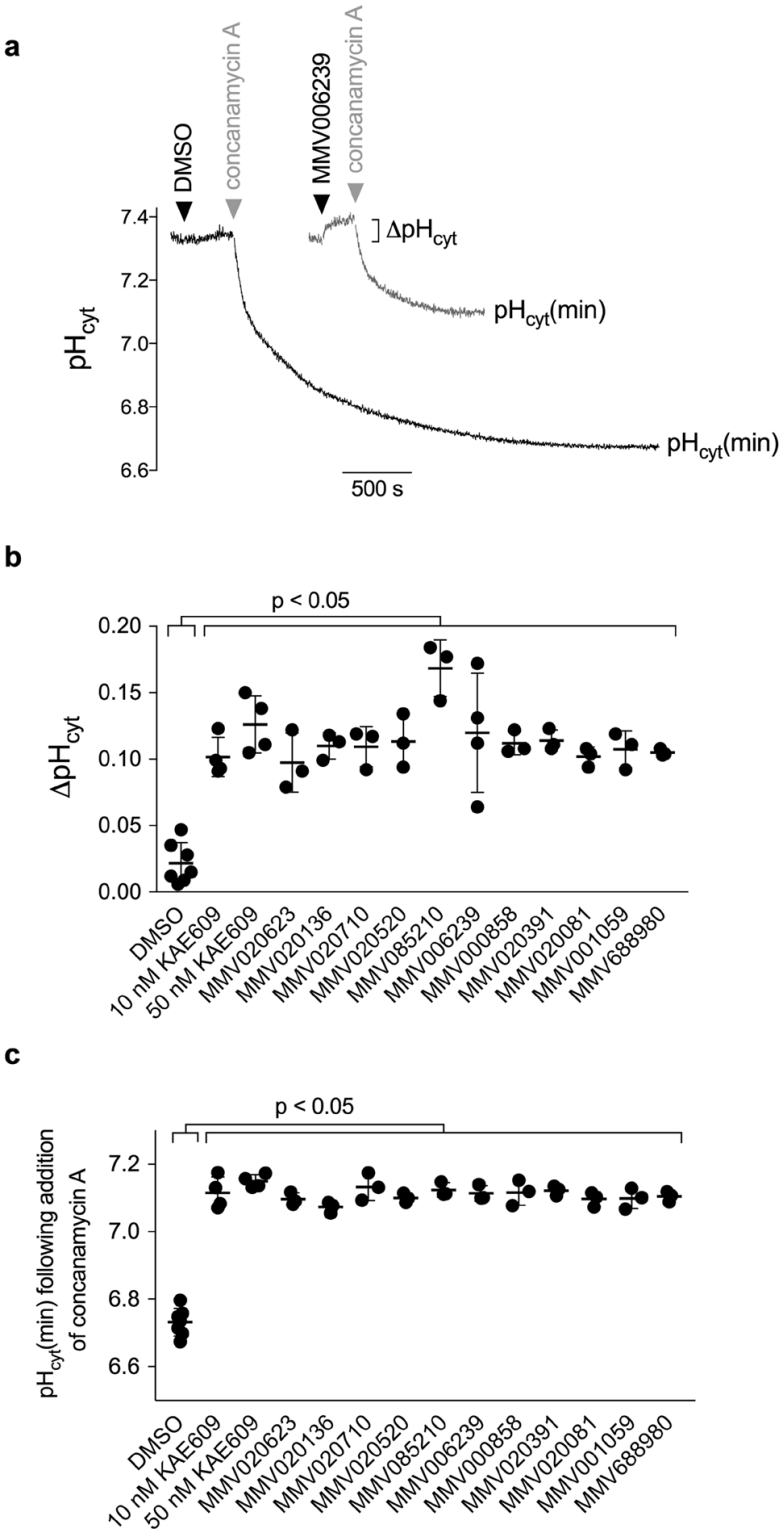
Effect of Pathogen Box compounds on pH_cyt_ of isolated asexual blood-stage 3D7 parasites. The BCECF-loaded parasites were suspended in physiological saline at 1–2 × 10^7^ cells/mL, and fluorescence was measured using a PerkinElmer LS 50B fluorimeter. (**a**) Traces showing the effects of DMSO (0.1% v/v; black trace) and MMV006239 (3.6 µM in DMSO; grey trace) on pH_cyt_. The addition of DMSO had no effect on pH_cyt_, whereas the addition of concanamycin A (100 nM; grey arrows) caused the cytosol to acidify to a pH well below that of the extracellular saline (pH 7.1). MMV006239 caused the cytosol to alkalinise, and reduced the acidification seen in response to concanamycin A. The results shown are from a single experiment and are representative of those obtained in at least three independent experiments. ∆pH_cyt_ denotes the magnitude of the pH_cyt_ increase observed following the addition of MMV006239. pH_cyt_(min) denotes the minimum pH reached following the addition of concanamycin A. (**b**) The magnitude of the alkalinisation observed following the addition of Pathogen Box compounds to parasites (i.e. ∆pH_cyt_). The Pathogen Box compounds (each tested at a concentration 15× the IC_50_ for inhibition of parasite proliferation; as listed below), and KAE609 (tested at 10 nM and 50 nM), caused a significant pH_cyt_ increase relative to the solvent control DMSO (0.1% v/v). (**c**) The magnitude of the acidification following treatment with concanamycin A (100 nM) in parasites exposed to Pathogen Box compounds, as indicated by pH_cyt_(min). The pH_cyt_(min) measured for concanamycin A-treated parasites exposed to Pathogen Box compounds (tested at a concentration 15× the IC_50_ for inhibition of parasite proliferation) was significantly higher than the pH_cyt_(min) measured for DMSO-exposed concanamycin A treated parasites. For panels (**b**) and (**c**) each symbol represents an individual measurement, with the mean pH_cyt_(min) averaged from the measurements made in at least three independent experiments indicated by the horizontal bars (shown ± SD). The concentrations tested were: 7.8 µM MMV020136; 1.47 µM MMV020710; 4.8 µM MMV020520; 3.6 µM MMV006239; 5.7 µM MMV000858; 4.2 µM MMV020391; 1.65 µM MMV020081; 10.8 µM MMV001059; 5.55 µM MMV020623; 16.8 µM MMV688980 and 24 µM MMV085210.

The PerkinElmer LS 50B fluorimeter provides a more sensitive measure of parasite pH_cyt_ than the 96-well plate reader, and it was used to quantify the effect of the eleven compounds on pH_cyt_ in BCECF-loaded isolated parasites. The resting pH_cyt_ in isolated parasites was 7.3 ± 0.1 (mean ± SD; n = 9) and the addition of DMSO (vehicle control; 0.1% v/v) was without effect (Fig. 3a). The addition of the V-type H^+^ ATPase inhibitor concanamycin A (100 nM) caused a pronounced cytosolic acidification. The addition of KAE609 (50 nM) caused a cytosolic alkalinisation and, furthermore, reduced the extent of the acidification seen in response to the addition of concanamycin A (Fig. 3b,c; Supplementary Fig. S3). Both effects have been attributed to KAE609 inhibiting the influx of H^+^ via PfATP4^3,4^. Each of the eleven Pathogen Box compounds tested had a similar effect to KAE609 on parasite pH_cyt_ when tested at a concentration 15× the IC_50_ for inhibition of parasite proliferation (see Fig. 3a for sample traces) and when tested at a concentration of 1 µM (Supplementary Fig. S3); i.e. the eleven Pathogen Box compounds caused significant alkalinisation of the parasite cytosol (p < 0.05, Fig. 3b), and significantly reduced the extent of the acidification observed following the addition of concanamycin A, when compared to the DMSO-exposed parasites (p < 0.05, Fig. 3c).

The 18 compounds causing optical effects in the 96-well SBFI assay were retested in the PerkinElmer LS 50B fluorimeter to determine whether they caused alkalinisation of the parasite cytosol in a similar manner to PfATP4-associated compounds. None of these 18 compounds induced alkalinisation of the parasite cytosol when added at a concentration of 1 µM; they were therefore discounted as potential PfATP4-associated compounds.

**Figure 4:**
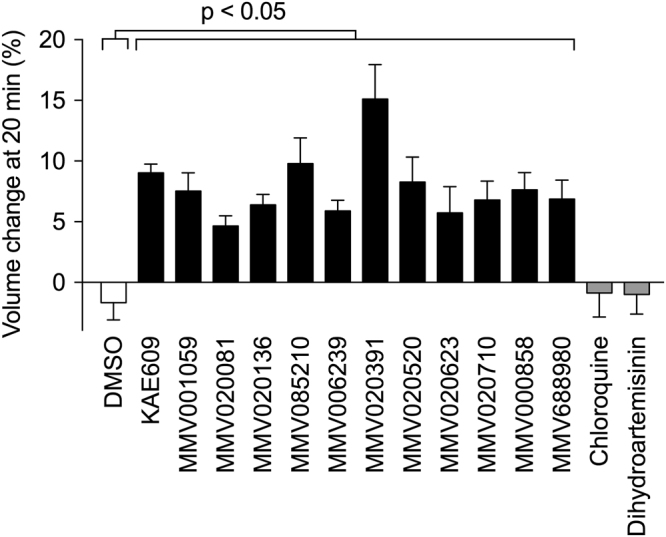
Effect of the 11 Na^+^-disrupting Pathogen Box compounds on the cell volume of isolated asexual blood-stage 3D7 parasites. Change in parasite volume (expressed as a percentage of the initial volume) after 20 min in the presence of KAE609 (10 nM, added in DMSO), selected Pathogen Box compounds, DMSO (0.1% v/v; included as a vehicle control), or the unrelated antimalarials dihydroartemisinin (30 nM, added in DMSO) and chloroquine (150 nM, added in water). The data are averaged from those obtained in at least three independent experiments. Error bars denote SD. The concentrations of the MMV compounds were as indicated in the legend to Fig. 3 (in each case equivalent to 15× the IC_50_ for inhibition of parasite proliferation).

The swelling of isolated parasites induced by each of the eleven compounds (tested at a concentration 15× IC_50_ for growth inhibition) was quantified using the Coulter Multisizer 4. All eleven induced significant parasite swelling over the course of a 20 min exposure (p < 0.05; compared to the DMSO control; Fig. 4). The MMV compounds caused parasite swelling of a magnitude similar to that induced by KAE609, with the exception of MMV020391, which caused significantly greater swelling than the KAE609 positive control (p < 0.05). The compound MMV688980, which failed to reach the ≥3% swelling threshold used in the initial volume screen when tested at a concentration of 5 µM, caused significant swelling when tested at a concentration of 16.8 µM (15× IC_50_ for growth inhibition; p < 0.05; compared to the DMSO control). By contrast, the unrelated antimalarials chloroquine (150 nM) and dihydroartemisinin (30 nM) were without significant effect on parasite volume (p > 0.05; compared to the DMSO control; Fig. 4).

**Figure 5:**
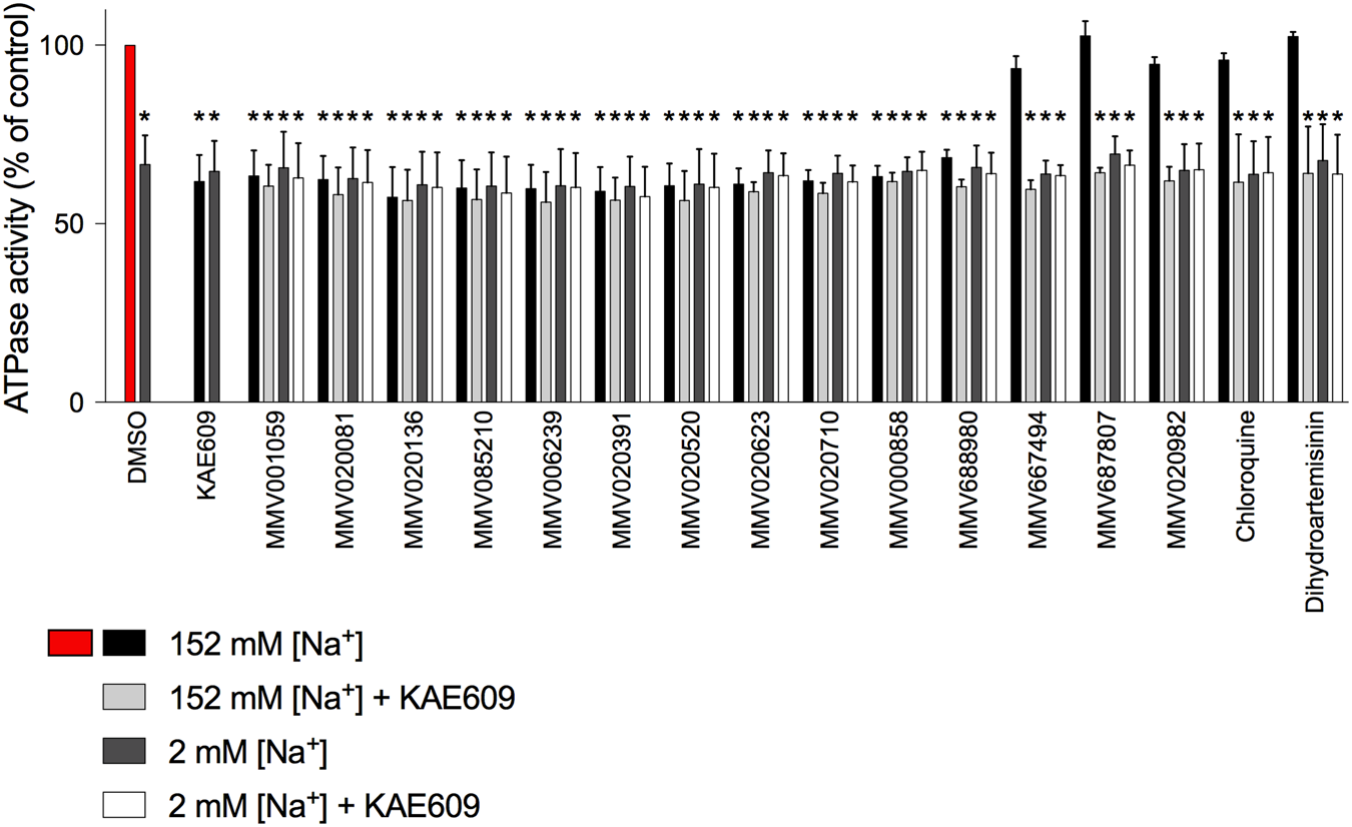
Effect of the 11 Na^+^-disrupting Pathogen Box compounds on membrane ATPase activity of asexual blood-stage 3D7 parasites. ATPase activity of parasite membrane preparations was measured both in high (152 mM) [Na^+^] medium and low (2 mM) [Na^+^] medium, and in the presence and absence of KAE609 (50 nM). The concentration of each of the Pathogen Box compounds was 1 µM. DMSO was tested at 0.1% v/v (as a vehicle control) and the antimalarial compounds dihydroartemisinin and chloroquine were tested at 30 nM (added in DMSO) and 150 nM (added in water), respectively. The data are averaged from at least four independent experiments, with ATPase activity shown as a percentage of that measured in the ‘DMSO control’ (i.e. in high (152 mM) [Na^+^] medium in the absence of any PfATP4 inhibitor; red bar). Error bars denote SD. The asterisks above the bars indicate statistical significance (*represents p < 0.05). Those values labelled with an asterisk are significantly different from that measured in the DMSO control.

In biochemical experiments on membrane fractions prepared from isolated parasites approximately one third of the membrane-associated ATPase activity was inhibited by KAE609 (50 nM; Fig. 5). Reduction of the Na^+^ concentration of the medium, from 152 mM to 2 mM similarly reduced the membrane-associated ATPase activity by approximately one third (Fig. 5). On addition of KAE609 (50 nM) to membranes in the low (2 mM) Na^+^ medium there was no further reduction of ATPase activity (Fig. 5; p > 0.05), consistent with KAE609 and Na^+^-depletion both eliminating the same (Na^+^-dependent) component of membrane-associated ATPase activity, as has been reported previously^4^. When added at a concentration of 1 μM, each of the eleven compounds identified as disrupting parasite [Na^+^]_cyt_ regulation inhibited membrane-associated ATPase activity to a similar extent to KAE609 (Fig. 5). The unrelated antimalarials chloroquine (150 nM) and dihydroartemisinin (30 nM), and three other randomly selected Pathogen Box compounds (MMV667494, MMV687807 and MMV020982; each tested at 1 μM) had no effect on ATPase activity when compared to the solvent control (0.1% v/v DMSO; p > 0.05; Fig. 5). For all eleven compounds there was no significant additional inhibition when KAE609 was added in addition to the MMV compound, and there was no significant additional inhibition when the [Na^+^] was reduced from 152 to 2 mM in the presence of each of the eleven compounds (p > 0.05; Fig. 5). Together, the data are consistent with all eleven compounds inhibiting the same Na^+^-dependent ATPase activity as is inhibited by the putative PfATP4 inhibitor KAE609.

**Figure 6:**
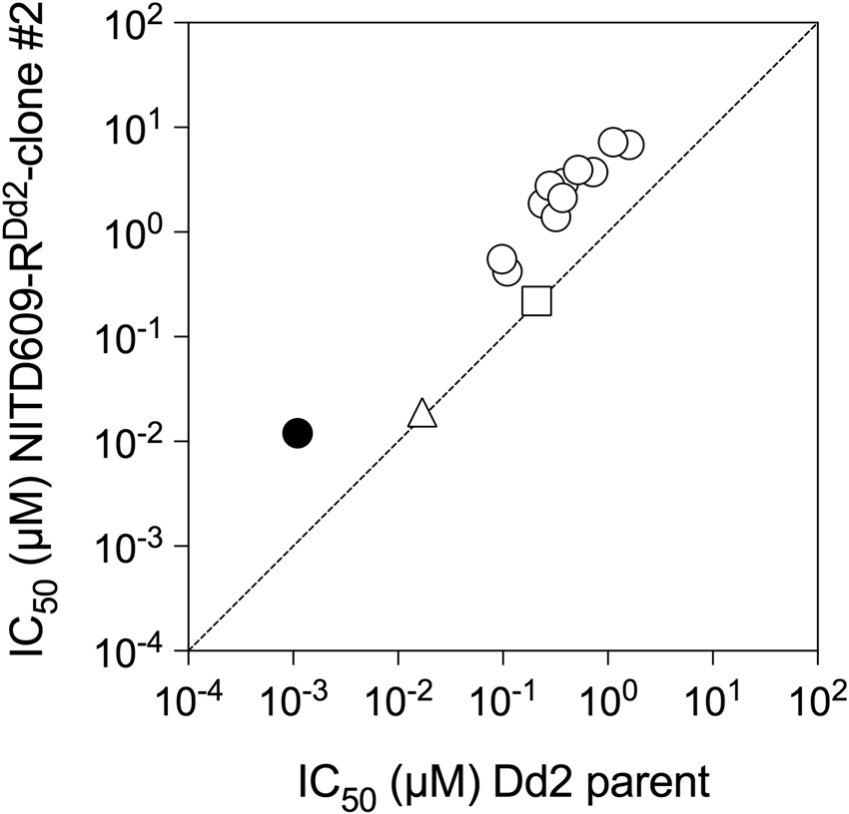
Cross-resistance of the spiroindolone resistant parasite line NITD609-R^Dd2^-clone#2 to the 11 Na^+^-disrupting Pathogen Box compounds. The IC_50_ for inhibition of the proliferation of the spiroindolone resistant NITD609-R^Dd2^-clone#2 parasite line (which carries two mutations in PfATP4: T418N and P990R) is plotted as a function of the IC_50_ for inhibition of the proliferation of the parental Dd2 line. The dashed line represents the line onto which compounds for which the two strains have identical IC_50_ values would fall. Those compounds to which the PfATP4 mutant (NITD609-R^Dd2^-clone#2) parasite line is resistant, relative to the parental line, fall above the dashed line. KAE609 is represented by the filled circle. The 11 PfATP4-associated compounds are represented by unfilled circles. The non-PfATP4-associated antimalarials artemisinin and chloroquine are represented by the triangle and square, respectively. The IC_50_ values for each compound, for both parasite lines, are listed in Table 2.

Finally, the eleven compounds were tested for the relative efficacies with which they inhibit the proliferation of a spiroindolone-resistant parasite line (NITD609-R^Dd2^-clone#2) and its Dd2 parent line^7^. The spiroindolone-resistant parasite line, carrying mutations in PfATP4, showed significant cross-resistance to all eleven compounds relative to the Dd2 parent parasites (p ≤ 0.001; Student t tests; Fig. 6; Table 2). By contrast, the spiroindolone-resistant parasite line showed the same sensitivity to artemisinin and chloroquine as its Dd2 parent line (Fig. 6).

## Discussion

**Figure 7:**
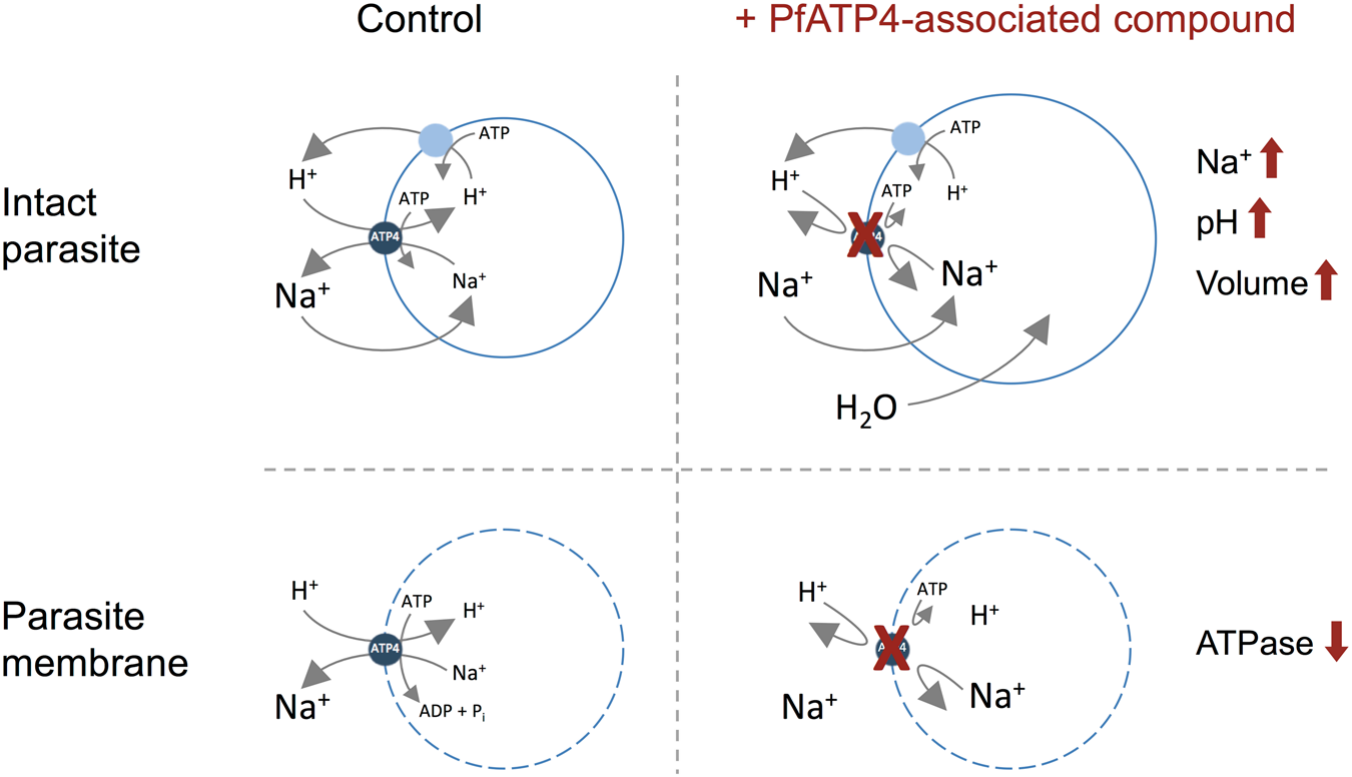
Schematic representation of the impact of PfATP4-associated compounds on the physiology and biochemistry of the malaria parasite. PfATP4 is postulated to function as a Na^+^/H^+^ ATPase, exporting Na^+^ from, and importing H^+^ into, the parasite. Inhibition of PfATP4 results in: an increase in parasite Na^+^ content and [Na^+^]_cyt_ resulting from the influx of Na^+^ down its electrochemical gradient, into the parasite, via as yet unidentified pathways; a cytosolic alkalinisation resulting from a cessation of the ‘acid loading’ associated with the import of H^+^ via PfATP4; an increase in parasite volume, resulting from the osmotic consequences of the increased Na^+^ content of the parasite; and the cessation of Na^+^-dependent ATPase activity in the parasite plasma membrane.

In this study the compounds comprising MMV’s Pathogen Box were screened for their ability to affect a number of key physiological parameters in isolated asexual blood-stage malaria parasites. Eleven of the 400 compounds tested were found to induce a progressive, immediate-onset increase in [Na^+^]_cyt_, similar to that induced by the antimalarial KAE609. Subsequent characterisation of these eleven compounds revealed that all shared the characteristics associated with a mechanism of action involving the ion-transporting ATPase, PfATP4. Specifically, all eleven compounds, in addition to causing an increase in [Na^+^]_cyt_: caused an alkalinisation of the parasite cytosol; reduced the magnitude of the cytosolic acidification seen on addition of the V-type H^+^ ATPase inhibitor concanamycin A; induced parasite swelling; inhibited the Na^+^-dependent, KAE609-sensitive membrane-associated ATPase activity; and showed reduced efficacy against parasites selected for resistance against KAE609 and carrying mutations in PfATP4. These features are summarised schematically in Fig. 7.

The eleven compounds are structurally diverse. One of the PfATP4-associated compounds, MMV006239, shares structural features in common with antimalarial spiroindolones (including KAE609 and the related spiroindolone NITD246^7^). However, the remaining ten compounds share little apparent structural similarity either to one another or to PfATP4-associated compounds identified previously^13^.

The simplest explanation consistent with the available data is that PfATP4 functions as a Na^+^-dependent (Na^+^ export/H^+^ import) ATPase, and that the eleven Pathogen Box compounds identified here exert their effects via a direct interaction with the protein, inhibiting the Na^+^ export/H^+^ import function, together with the associated Na^+^-dependent ATPase activity. Inhibition of Na^+^ export results in the observed increase in [Na^+^]_cyt_ as Na^+^ leaks into the parasite, down its electrochemical gradient via unknown pathways. The net influx of Na^+^ is accompanied by swelling of the parasite. Inhibition of H^+^ import, and the lifting of the associated ‘acid load’ results in the observed increase in the resting cytosolic pH, as well as the reduced acidification seen on inhibition of the primary H^+^ extrusion mechanism – the plasma membrane V-type H^+^ ATPase^12^ – by concanamycin A. The finding that parasites with mutations in PfATP4 show reduced sensitivity to growth inhibition by all eleven compounds is consistent with the compounds exerting their growth-inhibitory effect via an interaction with the PfATP4 protein.

All eleven of the compounds identified here as having a PfATP4-associated mechanism of action fell within the 125 compounds that had been included in the Pathogen Box on the basis of having been identified in whole cell phenotypic screens as potent inhibitors of the proliferation of asexual blood-stage *P*. *falciparum* parasites. None of the 275 compounds included in the Pathogen Box on the basis of their activity against other organisms showed the PfATP4-associated characteristics. The eleven compounds represent 9% of the 125 antimalarial compounds in the collection. This ‘hit rate’ is similar to that reported in a previous study^1^ in which it was found that 7% of the 400 compounds comprising the MMV Malaria Box showed an association with PfATP4. The 400 Malaria Box compounds, like the 125 antimalarial compounds included in the Pathogen Box, were selected on the basis of their activity in whole cell phenotypic screens against asexual blood-stage *P*. *falciparum* parasites. Both data sets therefore support the notion that 7–9% of the (structurally diverse) compounds identified in such screens share the same mechanism of action, exerting their antimalarial effect via an interaction with PfATP4. Why this one protein should be the target of such a high proportion of the antimalarial agents identified in whole cell screens is not clear. As noted previously^1^, this may reflect the fact that there are, in the parasite, a limited number of proteins that fulfil the requirements of a drug target, in being essential, accessible, and inhibitable by compounds at the low micromolar/submicromolar concentrations used in the phenotypic screens.

Most of the Pathogen Box compounds with activity against asexual blood-stage *P*. *falciparum* parasites have been tested for their effects on *Plasmodium* liver stages (*P*. *berghei* sporozoites; data are available via ChEMBL-NTD: https://www.ebi.ac.uk/chemblntd; accessed 5^th^ September 2017). The PfATP4-associated compounds identified here had variable, but typically poor, activity against *P*. *berghei* sporozoites, inhibiting their development by between 0 and 66% when tested at a concentration of 10 μM. This finding is consistent with previous studies that have noted the lack of liver stage activity of a spiroindolone^8^, a PfATP4-associated aminopyrazole^8^, and the PfATP4-associated compounds in the Malaria Box^14^.

The effects of the antiplasmodial Pathogen Box compounds on Stage V gametocytes have also been investigated (https://www.ebi.ac.uk/chemblntd; accessed 5^th^ September 2017). Ten of the eleven PfATP4-associated compounds identified in this study reportedly lacked potency against Stage V gametocytes, reducing their viability by ≤60% at a concentration of 10 μM. However, one of the eleven compounds, MMV020081, reduced the viability of Stage V gametocytes (and, in a separate study, Stage IV gametocytes^15^) with a submicromolar IC_50_. These data mirror previous findings with the PfATP4-associated Malaria Box compounds^14^, which were found to possess variable (and, on average, low) activity against late gametocytes (they were, however, more active against early gametocytes; discussed in ref.^1^). The activity of PfATP4-associated clinical lead compounds against sexual stage parasites has also been investigated using a variety of assays. Nanomolar concentrations of KAE609 were reported to reduce the viability of Stage II gametocytes, inhibit the development of late gametocytes from Stage II gametocytes, and inhibit the development of oocysts when added to the mosquito blood meal^16^. The lead pyrazoleamide PA92 was shown to inhibit the formation of (male and female) gametes from Stage V gametocytes at concentrations <100 nM^6^. The lead dihydroisoquinolone (+)-SJ733 was found to inhibit the transmission of *P*. *berghei* from mice to mosquitoes at doses lower than those required to kill asexual parasites^5^. Taken together, the available data indicate that PfATP4-associated compounds possess activity against a number of sexual stages, with mature gametocytes not being among the most susceptible.

The identification in this study of eleven new PfATP4-associated compounds increases the range of chemical structures known to show an association with PfATP4. Whether, and if so how, such a structurally diverse range of compounds can interact with, and inhibit, this protein is as yet unclear. Resolution of this issue awaits the successful heterologous expression and, ultimately, structure determination, for the protein.

In addition to identifying eleven PfATP4-associated compounds this study identified a number of other compounds that perturbed the pH_cyt_ and/or volume of the parasite. The molecular mechanism(s) underpinning these perturbations remain to be established.

## Methods

### Ethics statement

The use of human blood in this study was approved by the Australian National University Human Research Ethics Committee. The blood was provided by the Australian Red Cross Blood Service without disclosing the identities of the donors. The donors signed an agreement with the Australian Red Cross Blood Service permitting their blood to be used for medical research.

All methods used in this study were performed in accordance with the relevant guidelines and regulations.

### Parasite strains and culture

*Plasmodium falciparum* parasites of the 3D7 strain, the spiroindolone-resistant strain NITD609-R^Dd2^-clone#2^7^ (which carries two mutations in PfATP4: T418N and P990R) and its matched parental Dd2 strain, were cultured in human erythrocytes as described previously^17^. Parasites were synchronised by exposure to sorbitol (5% w/v)^18^ at least once in the 1–5 days prior to experimentation. With the exception of parasite proliferation assays, experiments were performed when the majority of parasites were at the mature-trophozoite stage (approximately 36–40 h post-invasion; range = 24–44 h post-invasion), as determined from Giemsa-stained smears.

### Compounds

The Pathogen Box was provided by the Medicines for Malaria Venture (MMV) and was comprised of 10 mM solutions in dimethyl sulfoxide (DMSO) of each of 400 compounds. Details of the compounds in the Pathogen Box are set out at CHEML-NTD (https://www.ebi.ac.uk/chemblntd; accessed 20^th^ April 2017) and the MMV Pathogen Box website (https://www.pathogenbox.org/; accessed 20^th^ April 2017). Each of the 10 mM solutions was diluted ten-fold in DMSO to yield 1 mM stocks which were dispensed as 10 µL aliquots into the individual wells of a 96-well plate for storage. The PfATP4-associated antimalarial KAE609 (cipargamin) and additional quantities of the Pathogen Box compounds MMV020136, MMV020710, MMV020520, MMV006239, MMV000858, MMV020391, MMV020081, MMV001059, MMV020623, MMV688980 and MMV085210 were obtained from MMV. Dihydroartemisinin was purchased from SelleckChem and chloroquine and artemisinin were purchased from Sigma. The MMV compounds, artemisinin and dihydroartemisinin were dissolved in DMSO; chloroquine was dissolved in water. All compound solutions were stored at −20 °C and thawed immediately prior to experimentation. Stock solutions were subjected to a maximum of 5× freeze-and-thaw cycles before being discarded.

### Isolation of trophozoite-stage parasites

Mature trophozoite-stage parasites were functionally isolated from their erythrocyte host cells by brief exposure of a parasitised cell culture (typically 4% haematocrit and 5% parasitaemia) to 0.05% w/v saponin (giving rise to a 0.005% w/v concentration of the active agent, sapogenin) as described elsewhere^19,20^. The saponin-treated cell suspensions (typically 50 mL) were centrifuged (550 × *g*, 5 min) and the sedimented parasites were washed three times in bicarbonate-free RPMI 1640 supplemented with 25 mM 4-(2-hydroxyethyl)piperazine-1-ethanesulfonic free acid (HEPES), 11 mM glucose (22 mM final concentration) and 200 µM hypoxanthine (12 000 × *g*, 30 s). The isolated parasites were then suspended in either supplemented bicarbonate-free RPMI or physiological saline (125 mM NaCl, 5 mM KCl, 1 mM MgCl_2_, 25 mM HEPES, 20 mM glucose; pH 7.1 with NaOH) and incubated at 37 °C prior to beginning each experiment. Parasites isolated from their erythrocyte host using saponin maintain transmembrane ion gradients^3,4,21^ and an inwardly-negative membrane potential^22^.

### Parasite [Na^+^]_cyt_ and pH_cyt_ measurements

For measurements of [Na^+^]_cyt_ isolated parasites were loaded with the acetoxymethyl (AM) ester of the Na^+^-sensitive fluorescent dye sodium-binding benzofuran isophthalate (SBFI, Life Technologies) as described elsewhere^4^, and suspended in physiological saline at a density of 1–2 × 10^7^ parasites/mL. For the initial screen of the Pathogen Box the fluorescent signal from SBFI-loaded parasites was measured in a Tecan fluorescence spectrometer plate-reader. In subsequent experiments with those compounds identified in the initial screen as ‘hits’, measurements with greater sensitivity were made using a PerkinElmer LS 50B fluorescence spectrometer with a dual excitation Fast Filter accessory. For measurements on both instruments, SBFI-loaded parasites were excited at wavelengths of 340 nm and 380 nm, and the emission was recorded at 515 nm. The ratio of the fluorescence intensity measured at each of the excitation wavelengths (340 nm/380 nm) is indicative of parasite [Na^+^]_cyt_. Measurements performed in the PerkinElmer LS 50B fluorescence spectrometer (but not those obtained using the plate reader) were calibrated, such that measurements of the fluorescence ratio yielded an estimate of parasite [Na^+^]_cyt_, as described previously^4^.

For measurements of pH_cyt_, isolated parasites were loaded with the AM ester of the pH-sensitive fluorescent dye 2′,7′-Bis(2-carboxyethyl)−5(6)-carboxyfluorescein (BCECF, Life Technologies) as described elsewhere^1,4^, then suspended in physiological saline at a density of 1–2 × 10^7^ parasites/mL. For the initial measurements in the Tecan plate-reader, as well as subsequent measurements in the PerkinElmer LS 50B fluorescence spectrometer, the excitation wavelengths were 440 nm and 495 nm, and emission was recorded at a wavelength of 520 nm. The ratio of the fluorescence intensity measured at each of the excitation wavelengths (495 nm/440 nm) provides a measure of parasite pH_cyt_. The fluorescence ratio measurements made in the PerkinElmer LS 50B fluorescence spectrometer (but not those obtained using the plate-reader) were calibrated to enable estimates of pH_cyt_ as described previously^12^.

In the initial screen of the Pathogen Box for compounds that disrupted [Na^+^]_cyt_ and/or pH_cyt_, using the Tecan fluorescence plate-reader, fluorescence intensity was measured for 20–40 min with a measurement taken every 15–45 s. Compounds were loaded into individual wells of a 96-well plate at 40× the required final concentration in 5 µL of physiological saline. A 195 µL aliquot of dye-loaded parasite suspension was then added into each well, 5–30 s prior to measuring the fluorescence intensity, giving a final cell concentration of 1–2 × 10^7^ parasites/mL. For the purpose of the initial screens a 1 µM concentration was used for each compound. The final concentration of DMSO in each well was 0.1% v/v.

The ‘vehicle control’ for both [Na^+^]_cyt_ and [pH]_cyt_ measurements was 0.1% v/v DMSO. For [Na^+^]_cyt_ measurements, the positive controls were the spiroindolone KAE609 (50 nM), which causes parasite [Na^+^]_cyt_ to increase (by inhibiting the active extrusion of Na^+^ via PfATP4^4,9^), and the Na^+^-ionophore gramicidin (5 µM), which permeabilises the parasite membrane to Na^+^, resulting in a rapid influx of Na^+^^4^. For pH_cyt_ measurements, the V-type H^+^ ATPase inhibitor concanamycin A (100 nM), which causes a rapid acidification of the parasite cytosol^4,12^, was included as a positive control. For both Na^+^ and pH measurements, 15–35 Pathogen Box compounds were tested per 96-well plate. Each row of the 96-well plate held five Pathogen Box compounds and the appropriate set of control compounds.

### Parasite volume measurements

The Pathogen Box compounds were screened for effects on the volume of isolated trophozoites using a Beckman Coulter Multisizer 4 with a 100 µm ‘Aperture tube’, as described elsewhere^10,23^. Isolated trophozoites were suspended at 4 × 10^5^ parasites/mL in glucose-containing physiological saline and incubated at 37 °C for 15–20 min in the absence of any compound to allow the parasites to recover from the saponin-isolation procedure. Parasite volume was measured at time-zero (immediately prior to the addition of compound) and 20 min after the addition of compound. Each measurement consisted of approximately 20 000 pulses, with each pulse corresponding to the passage of a cell through the aperture. The resulting parasite volume population data was fitted with a log Gaussian distribution and the median volume of the population thereby determined.

The volume measurements are relatively low-throughput (compared to the 96 well plate assays) and a screening strategy was therefore devised as follows:

1. Those compounds that were found in the initial [Na^+^]_cyt_ and/or pH_cyt_ screens to perturb parasite ion homeostasis were tested individually (at a concentration of 5 µM) for their effect on parasite volume.
2. Those compounds that were found in the initial [Na^+^]_cyt_ and pH_cyt_ screens *not* to perturb parasite ion homeostasis were combined in ‘groups of five’ which were then tested together; i.e. the parasites were exposed to the five compounds at the same time. Each of the five compounds was present at a concentration of 1 µM.
3. For any group-of-five compounds found to perturb the parasite volume by ≥3% in the initial measurement, the experiment was repeated a second time to confirm the original observation.
4. For those group-of-five compounds that were confirmed as perturbing parasite volume by ≥3%, each of the five compounds was tested individually for its effect on parasite volume when added at a concentration of 5 µM.
5. For any of the individual compounds from a group-of-five found to perturb the parasite volume by ≥3% in the initial measurement, the experiment was repeated a second time to confirm the finding.

The application of the strategy is illustrated schematically in Supplementary Fig. S2. The ‘vehicle control’ for volume measurements was 0.5% v/v DMSO for the initial screening process and 0.1% v/v DMSO for all other measurements.

### Membrane ATPase activity assays

Membranes were prepared as described previously^4^, with minor modifications. Briefly, saponin-isolated trophozoites were lysed by first sedimenting the cells by centrifugation (12,000 × *g*, 30 s) then resuspending them in ice-cold sterile deionised H_2_O (containing a 1/500 dilution of Protease Inhibitor Cocktail Set III; Merck Millipore), thereby inducing cell lysis. The crude membrane preparation was then washed three times in ice-cold water (containing the protease inhibitors for the first two washes; 14,000 × *g*, 10 min) at 4 °C and the protein content measured using a modified Bradford Assay^24^.

ATPase activity was measured using the PiColorLock Gold Phosphate Detection System (Innova Biosciences) in a 96-well plate format. The reaction mixture in each well consisted of: (1) a pH 7.4 solution containing KCl, MgCl_2_ and Tris-HCl (yielding final concentrations of 20 mM, 2 mM and 50 mM, respectively); (2) parasite membrane (at a final concentration of 50 μg protein/mL); (3) NaCl or choline chloride (yielding a final concentration of 150 mM); and (4) ATP (Na_2_ATP.3H_2_O; MP Biomedicals; added last at a final concentration of 1 mM to initiate the reaction). It was necessary to use Na_2_ATP as the commercially available K^+^ and Mg^2+^ salts of ATP both have significant phosphate contamination, which interferes with the (phosphate-based) ATPase assay. The use of the Na_2_ATP salt (1 mM) resulted in a 2 mM concentration of Na^+^ in the reaction solution.

Following a 10 min incubation at 37 °C, 100 µL of each reaction mixture was added to 25 µL of the colourimetric reagent (‘Gold mix’), which terminated the reaction. ‘Stabiliser’ (10 μL) was added 2.5 min later and the plate was then incubated for a further 1 h before absorbance was measured at 635 nm. Blank values (from wells containing only water, Gold mix and stabiliser) and control values (from wells containing all components, but for which ATP was not added until after the membrane was exposed to Gold mix) were subtracted from the data.

The effects of the compounds of interest on ATPase activity were assessed both in high-Na^+^ medium (containing 152 mM NaCl) and low-Na^+^ medium (containing just the 2 mM Na^+^ originating from the Na_2_ATP salt), and in the presence and absence of the putative PfATP4 inhibitor KAE609 (50 nM). The final concentration of DMSO in each well was 0.2% v/v.

### Parasite proliferation assays

The effect of compounds of interest on the proliferation of *P*. *falciparum* parasites was measured over 72 h with a starting parasitaemia of 1% ring-stage parasites and a haematocrit of 1%. Parasite proliferation was monitored using the fluorescent DNA-intercalating dye SYBR Safe as described previously^25^.

### Statistical analysis

Unless stated otherwise, statistical comparisons were made using one-way Analysis of Variance (ANOVA) on pre-normalised data, blocked by experiment. The ANOVA was followed by Bonferroni’s post-hoc test when appropriate.

The percentage coefficient of variation for the parasite volume estimates made in the course of the screen of the Pathogen Box compounds was calculated by dividing the average of the standard deviations for the parasite volume estimates made in replicate experiments by the mean value for the parasite volume, then multiplying this value by 100. The inter-assay percentage coefficient of variation for the parasite volume screening assay was determined by calculating the mean of the percentage coefficients of variation for the positive control (parasite volume following 20 min exposure to KAE609) and the negative control (parasite volume following 20 min exposure to DMSO). This yielded a value of 12%.

For the purpose of the Pathogen Box screens the [Na^+^]_cyt_ and pH_cyt_ assays were used on a qualitative basis, thereby precluding the calculation of an inter-assay coefficient of variation.

### Data availability statement

The data generated in the course of this study, and the Standard Operating Procedures used throughout, are available from the corresponding author on reasonable request.

## Electronic supplementary material

Supplementary Information
