# Supplementary material for: Diverse antimalarials from whole-cell phenotypic screens disrupt malaria parasite ion and volume homeostasis
Source: Sci Rep. 2018 Jun 11;8:8795. doi: 10.1038/s41598-018-26819-1 (PMC5995868; doi:10.1038/s41598-018-26819-1)
Supplement: Supplementary file 1 — Supplementary Information [file 41598_2018_26819_MOESM1_ESM.pdf]

Supplementary Information

Diverse antimalarials from whole-cell phenotypic screens disrupt malaria parasite ion and volume homeostasis

Adelaide S. M. Dennis, James E. O. Rosling, Adele M. Lehane*, Kiaran Kirk*

*Corresponding authors (email: adele.lehane@anu.edu.au, kiaran.kirk@anu.edu.au)

Supplementary Table S1. Pathogen Box compounds that caused a cytosolic acidification in isolated BCECF-loaded parasites when added at 1 µM (n = 1), and/or had an effect on isolated parasite volume when added at 5 µM (n = 2). Compounds that caused an effect on $[Na^+]_{cyt}$ in isolated SBFI-loaded parasites are listed in Table 1.

Compound Identifier	Location in the Pathogen Box	Anti-pathogen activity	Caused a decrease in pH_{cyt}	Change in parasite volume (%)
MMV676501	Plate A, A9	Tuberculosis	Yes	-4
MMV102872	Plate A, B10	Tuberculosis	Yes	-8
MMV676477	Plate A, B11	Tuberculosis	Yes	-4
MMV676558	Plate A, G7	Tuberculosis	Yes	-
MMV688991	Plate B, G3	Reference compound Nitazoxanide	Yes	-6
MMV687807	Plate C, F8	Tuberculosis [a]	Yes	-4
MMV659004	Plate D, E5	Kinetoplastids [b]	Yes	+3
MMV688345	Plate E, D7	Toxoplasmosis	Yes	-
MMV021013	Plate E, G4	Tuberculosis	Yes	-
MMV658988	Plate E, G8	Kinetoplastids [b]	Yes	-
MMV002817	Plate B, E6	Onchocerciasis	No	-20
MMV019993	Plate E, A11	Malaria	No	-13
MMV407834	Plate E, E9	Malaria	No	-12
MMV020512	Plate B, A10	Malaria	No	-10
MMV687812	Plate D, F11	Tuberculosis	No	-7
MMV688978	Plate E, H5	Reference compound Auranofin	No	-7
MMV676476	Plate A, F7	Tuberculosis	No	-5
MMV676412	Plate A, A11	Tuberculosis	No	-5
MMV153413	Plate E, E11	Tuberculosis	No	-5
MMV676524	Plate E, C10	Tuberculosis	No	-4
MMV012074	Plate B, B2	Tuberculosis	No	-3
MMV011903	Plate B, A7	Malaria	No	+3
MMV085210 [c]	Plate B, F11	Malaria	No	+3
MMV020623 [c]	Plate B, A9	Malaria	No	+4
MMV001059 [c]	Plate D, G3	Malaria	No	+4
MMV020136 [c]	Plate B, C7	Malaria	No	+5
MMV020710 [c]	Plate B, C8	Malaria	No	+5
MMV000858 [c]	Plate B, G8	Malaria	No	+5
MMV020520 [c]	Plate B, D8	Malaria	No	+6
MMV006239 [c]	Plate B, G7	Malaria	No	+6
MMV020081 [c]	Plate D, D3	Malaria	No	+6
MMV020391 [c]	Plate B, H9	Malaria	No	+11

[a] The anti-tuberculosis compound MMV687807 caused a decrease in parasite pH_{cyt} that was similar in magnitude to that seen for the control compound concanamycin A. All other pH-disrupting compounds listed here caused an acidification of the parasite cytosol that was less pronounced than that observed for concanamycin A.

[b] Compounds MMV659004 and MMV658988 are 2-Pyridyl-4-aminopyrimidines with asexual stage antiplasmodial activity[1].

[c] These compounds caused an increase in $[Na^+]_{cyt}$ and are also listed in Table 1.

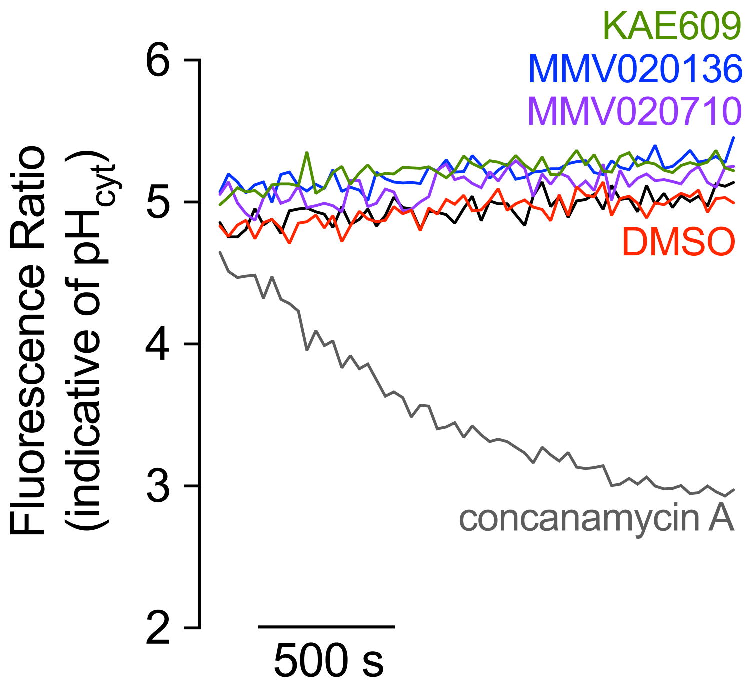

Supplementary Figure S1. Trace showing Pathogen Box compounds causing a slight alkalinisation of the parasite cytosol in the 96-well plate assay. Trace showing the effects of concanamycin A (100 nM, grey trace), KAE609 (50 nM, green trace), 0.1% v/v DMSO (vehicle control, red trace) on pH_{cyt} in isolated BCECF-loaded parasites. Pathogen Box compounds, represented by a blue trace for MMV020136 and a purple trace for MMV020710 (each at a concentration of 1 µM) caused an increase in parasite $[Na^+]_{cyt}$, whereas the compound represented by the black trace (MMV020517, 1 µM) had no effect on parasite $[Na^+]_{cyt}$. Parasites were suspended in physiological saline at 1-2 × 10^7 cells/mL. Fluorescence was measured using the Tecan fluorescence plate reader.

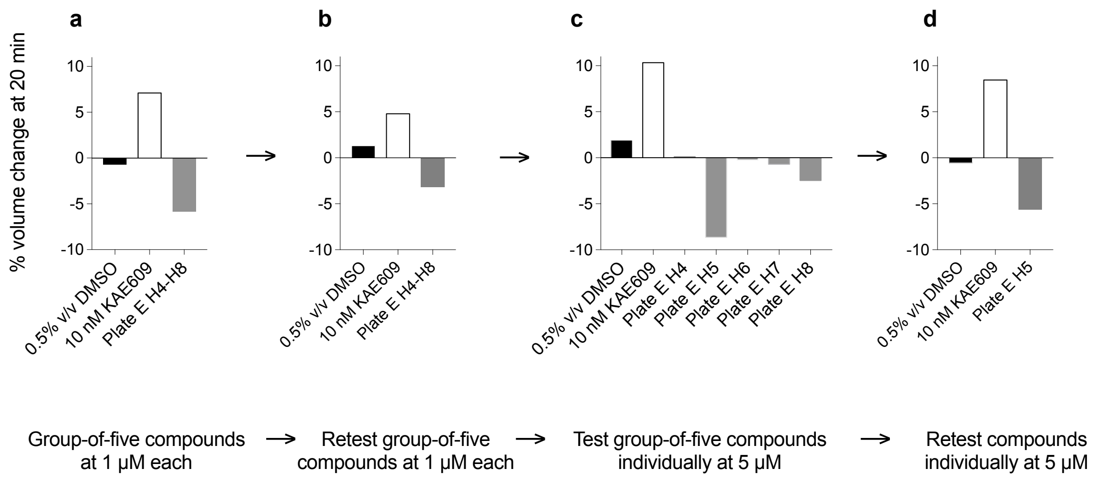

Supplementary Figure S2. Screening strategy used to identify Pathogen Box compounds that have an effect on parasite volume. (**a**) Isolated parasites were suspended in physiological saline and exposed either to the vehicle control (0.5% v/v DMSO), to KAE609 (10 nM in DMSO) or to groups of five Pathogen Box compounds, each at a concentration of 1 µM. Parasite volume was measured at t = 0 and 20 min using a Coulter Multisizer 4, and the % volume change at 20 min thereby determined. (**b**) Groups of five compounds that caused a volume change of ≥ 3% when added to isolated parasites, each at a concentration of 1 µM (in (**a**)) were tested a second time. (**c**) The groups of five compounds that caused a volume change of ≥ 3% in both of the two replicates (i.e. in (**a**) and (**b**)) were tested individually, each at a concentration of 5 µM. (**d**) Each individual compound found to disrupt parasite volume by ≥ 3% at 5 µM (in (**c**)) was tested a second time to confirm the result.

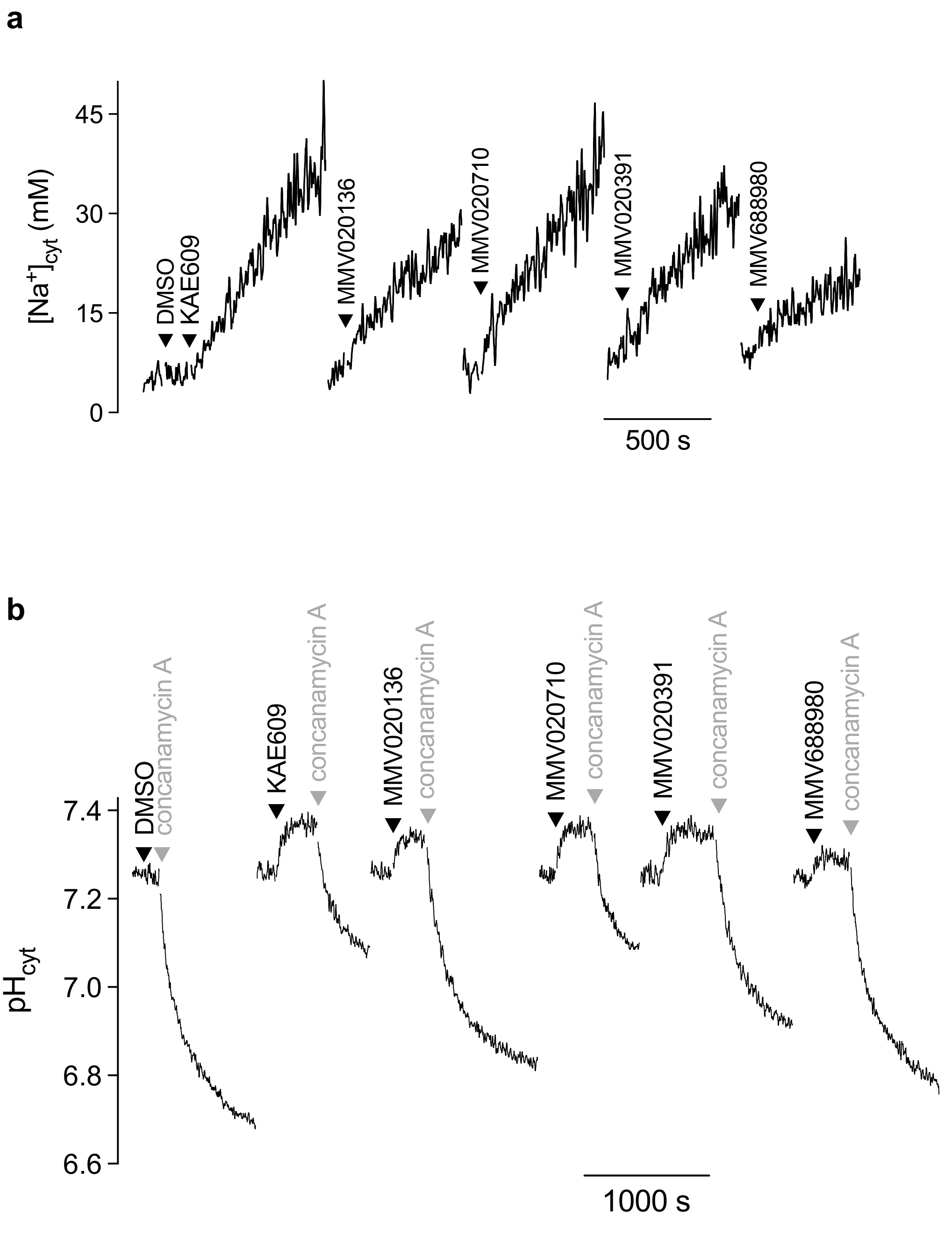

Supplementary Figure S3. The effect of representative Pathogen Box compounds (each tested at 1 µM) on the $[Na^+]_{cyt}$ and pH_{cyt} of asexual blood-stage 3D7 parasites. (**a**) Traces showing the effects of selected compounds on $[Na^+]_{cyt}$ in isolated SBFI-loaded parasites. DMSO (0.1% v/v), added as a solvent control, had no effect on parasite $[Na^+]_{cyt}$, whereas the addition of KAE609 (50 nM) caused an immediate-onset increase in $[Na^+]_{cyt}$. The addition of the Pathogen Box compounds

MMV020136, MMV020710, MMV020391 or MMV688980 (each added at a concentration of 1 µM) caused parasite $[Na^+]_{cyt}$ to increase. The results shown are from a single experiment. (**b**) Representative traces showing the effects of selected compounds on pH_{cyt} in isolated BCECF-loaded parasites. The addition of concanamycin A (100 nM; grey arrows) caused the cytosol to acidify to a pH below that of the extracellular saline (pH 7.1), whereas the addition of DMSO (0.1% v/v; solvent control) had no effect on pH_{cyt}. KAE609 (10 nM in DMSO) caused the cytosol to alkalinise, and reduced the acidification seen in response to the addition of concanamycin A. The Pathogen Box compounds - MMV020136, MMV020710, MMV020391, and MMV688980 (each tested at 1 µM) - had a similar effect, causing a cytosolic alkalinisation and reducing the concanamycin A-induced acidification. The results shown are from a single experiment and are representative of those obtained in two independent experiments. For (**a**) and (**b**) parasites were suspended in physiological saline at 1-2 × 10^7 cells/mL, and fluorescence intensity was measured using a PerkinElmer LS 50B fluorimeter.

Reference

1. Duffy, S., Sykes, M. L., Jones, A. J., Shelper, T. B., Simpson, M. *et al.* Screening the Medicines for Malaria Venture Pathogen Box across multiple pathogens reclassifies starting points for open-source drug discovery. *Antimicrob Agents Chemother* **61**, e00379-17 (2017).
